# Factors affecting the clinical outcomes including patient satisfaction after Oxford unicompartmental knee arthroplasty: a retrospective study

**DOI:** 10.1186/s42836-020-00038-4

**Published:** 2020-07-09

**Authors:** Yang Chen, Xinyu Fang, Zida Huang, Wenbo Li, Wenming Zhang

**Affiliations:** Department of Orthopedic Surgery, The First Affiliated Hospital of Fujian Medical University, Fuzhou, 350004 China

**Keywords:** Unicompartmental knee arthroplasty, Numerical rating Scalescore, Knee society score, Patient satisfaction

## Abstract

**Background:**

Oxford unicompartmental knee arthroplasty (UKA) is widely used for treating patients with unicompartmental knee diseases. However, the factors affecting the outcomes of and patients’ satisfaction with Oxford UKA remain controversial. The aims of this study were to evaluate the clinical and radiological outcomes, including patient satisfaction, after Oxford UKA and identify the influencing factors related to patients’ satisfaction.

**Methods:**

We retrospectively analyzed the data of patients who underwent UKA in our medical institution from 2013 to 2018. Demographic information, clinical scores, patient satisfaction and imaging findings were recorded. The patients were followed up for at least 1 year. Multivariate Logistic regression analysis was performed to identify influencing factors related to clinical outcomes including patient satisfaction.

**Results:**

A total of 80 patients (involving 87 knees) were included. The Numerical Rating Scale (NRS) scores and Knee Society Scores (KSS) were significantly improved after operation as compared to preoperative scores (6.0 vs. 1.0, *P* < 0.001; 113.0 ± 27.2 vs. 167.2 ± 23.9, *P* < 0.001), and the varus deformity was corrected (181.0 vs. 176.0, *P* < 0.05). Six patients (7.5%) developed postoperative complications, and no case suffered from prosthesis loosening. The time since operation (TSO) and angle E were found to be predictors of KSS improvement (*P* = 0.009; *P* = 0.024). The postoperative KSS and angle E were found to be predictors of patient satisfaction (*P* = 0.001; *P* = 0.032).

**Conclusion:**

Oxford UKA can improve the NRS score and KSS and correct varus deformities. A shorter TSO and smaller angle E are indicators of a greater KSS improvement. A higher KSS and smaller angle E are indicative of higher patient satisfaction.

## Background

Knee osteoarthritis (OA) is the most common cause of knee pain and dysfunction in elderly people. Due to population aging, the incidence of knee OA has been on a substantial rise. The incidence of knee OA is 28% in people over 45 years old [1] and is as high as 40% in people over 70 years old [2]. The common treatments of knee OA consist of conservative treatment, such as physiotherapy and pharmacotherapy, and surgery, which includes arthroscopy, high tibial osteotomy (HTO), unicompartmental knee arthroplasty (UKA) and total knee arthroplasty (TKA) [2]. The treatment is mostly selected according to the degree of disease progression.

Currently, it is generally accepted that UKA is an alternative treatment for unicompartmental OA of the knee. More than 60 years have passed since Dr. McKeever designed the first unicompartmental knee prosthesis and performed the first UKA in the early 1950s [3]. Since then, the prosthesis has been constantly improved thank to the effort of a great many orthopedic surgeons and engineers. In 1978, Goodfellow and O’Connor designed the first Oxford unicompartmental knee prosthesis [4], which is composed of three parts: a spherical femoral component, a flat tibial component, and a movable polyethylene bearing designed to perfectly match the femoral component. This prosthesis is a typical design used in mobile-bearing UKA.

In recent years, the value of UKA has been increasingly recognized by orthopedic surgeons. The advantages of UKA over total knee arthroplasty (TKA) include less trauma, shorter operative time, less intraoperative bleeding, faster recovery and better proprioception of the patient [5, 6]. In the past, UKA had strict indications, but now surgeons have performed the procedure on patients of younger age, for spontaneous osteonecrosis of the knee (SONK) and anterior cruciate ligament (ACL) injury [7–9]. According to literature, the Knee Society Score (KSS) and survival of prostheses were improved after UKA, and the reoperation rate has dropped over the past few decades [10]. Unfortunately, previous studies showed that the efficacy of UKA has been evaluated in terms of a variety of objective indicators and scores, not in light of the subjective feeling of patients [11, 12]. The aim of this study was to evaluate the clinical and radiological outcomes after Oxford UKA in our center. Demographic data, surgical information and clinical scores were analyzed to identify the influencing factors related to clinical outcomes.

## Materials and methods

### Patient selection

We retrospectively analyzed the data from patients who underwent UKA in our medical institution between November 2013 and December 2018.The inclusion criteria included: (1) UKA due to medial compartment knee OA or medial femoral condyle SONK, (2) preoperative imaging results indicative of a lesion that did not involve the lateral compartment, and (3) preoperative evaluation showing the anterior and posterior cruciate ligaments and the collateral ligaments were intact. The exclusion criteria were: (1) the presence of other inflammatory knee diseases, meniscus injury or traumatic arthritis, (2) incomplete medical records, and (3) a follow-up period less than 1 year.

### Operation and postoperative rehabilitation

All patients received an Oxford unicompartmental knee prosthesis (Zimmer Biomet, Warsaw, IN), and all the operations were performed by the same experienced orthopedic surgeon. The standard procedure for Oxford UKAs was done. Mixed analgesics were injected into the soft tissues around the knee before the incision was closed. Prophylactic antibiotics were administered 30 min before the operation. The drainage tubes were removed within 24 h after the operation. Routine anticoagulant and analgesic therapy was given. Knee flexion and extension exercises were started on the first day after the operation, and patients were instructed to practice partial weight-bearing walking with walking aids appropriate for their specific conditions.

The time interval between the date of operation and January 2020 was defined as the time since operation (TSO). The longer the TSO, the more the time that elapsed since the operation and vice versa.

### Clinical review

Demographic data such as age, sex, BMI and operated side were recorded. The Numerical Rating Scale (NRS) score [13, 14] and KSS [15] were recorded before, 3 days, 1, 3, 6, 12 month(s) after operation and, thereafter, annually.

### Patient satisfaction

A 6-level Likert scale [16] was used to rate patient satisfaction, with excellent, very good, good, fair, poor and terrible result, respectively, listed as 1, 2, 3, 4, 5 and 6 point(s). Patients who reported 1 to 3 point(s) were included in the satisfaction group, and those who gave 4 to 6 points were included in the dissatisfaction group. Patient satisfaction was recorded at each follow-up.

### Radiographical review

**Fig. 1:**
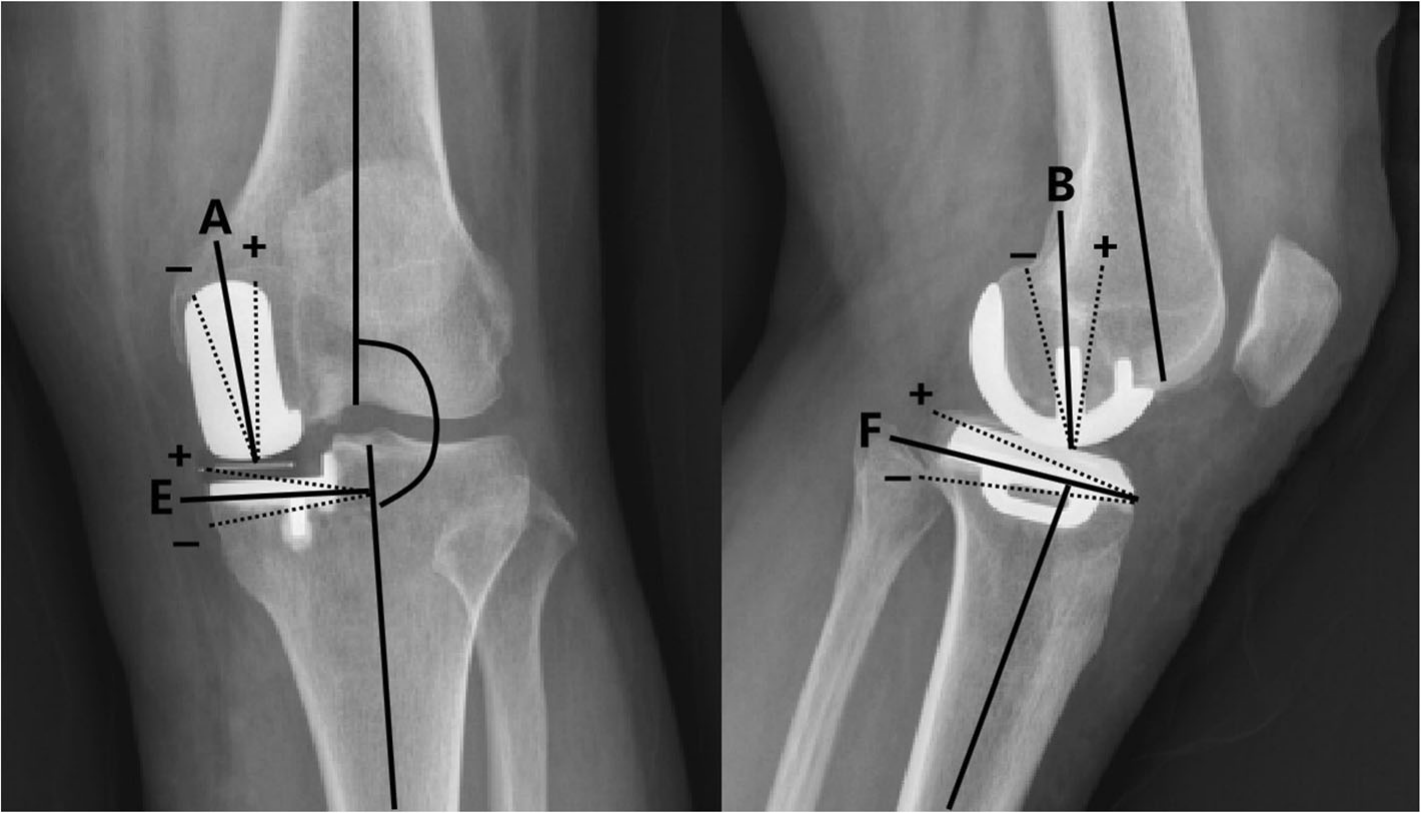
Measurement of FTA and component alignments. FTA, femorotibial angle. Angle A defined as femoral component varus/valgus angle; angle B was flexion/extension angle of femoral component; angle E defined as tibial component varus/valgus angle; angle F was posterior tibial component slope

X-rays were taken before the operation and at each follow-up. The positioning and alignment of the implants were evaluated according to the recommendations of the implant manufacturer (Zimmer Biomet) [17]. The indicators included angle A, angle B, angle E, angle F, the femorotibial angle (FTA), and the degree of overhang, loosening and dislocation of the prosthesis (Fig. 1). Angle A was defined as varus/valgus angle of the femoral component (10° varus to 10° valgus). Angle B was defined as the flexion/extension angle of the femoral component (10° flexion to 5° extension). Angle E was defined as the varus/valgus angle of the tibial component (5° varus to 5° valgus). Angle F was defined as the posterior slope of the tibial component (2° to 12°). Excessive deviation was defined as the presence of angles exceeding the allowable deviation range. The FTA was defined as the lateral angle formed by the long axes of the femoral and tibial shafts. Prosthesis overhang was present when the tibial prosthesis exceeded the medial and posterior edges of the tibial plateau by more than 2 mm. A progressive radiolucent line around the prosthesis with a width greater than 2 mm indicated the existence of prosthesis loosening.

### Statistical analysis

The Kolmogorov-Smirnov test was used to assess the normality of the data for continuous variables. Continuous variables with normal distribution were expressed as the means ± standard deviations (SDs) and 95% confidence intervals (CIs). The remaining variables were expressed as the medians and quartiles. The categorical variables were expressed as the sums and percentages. The *t*-test was used to compare the differences between the two groups for the continuous variables with normal distribution, and the Mann-Whitney U test was employed for those with non-normal distribution. The categorical data were compared by using the McNemar X^2^ test or Fisher’s exact test. The significant predictors emerging from the univariate analysis were selected, and a forward stepwise procedure in a multivariate logistic regression model was performed. The results of the regression analyses were presented as the odds ratios (ORs) with 95% CIs. Statistical analyses were conducted by using SPSS for Windows (Version 25, IBM, Armonk, NY, USA). Differences were considered to be statistically significant at *P* < 0.05.

## Results

A total of 97 patients (104 knees) underwent UKA in our medical institution from November 2013 to December 2018. Eighty of them (87 knees), including 22 males (25 knees) and 58 females (62 knees) were included and17 patients were excluded because they were lost to follow-up. On average, 80 patients were aged 64.5 ± 7.4 years (95% CI, 62.9–66.1 years) and had a BMI of 26.1 ± 3.9 kg/m^2^ (95% CI, 25.2–26.9 kg/m^2^), with female patients being significantly more than male ones (*P* < 0.001). Thirty-seven patients had preoperative complications, including hypertension, diabetes, cardiovascular diseases, and hepatic and renal dysfunction. All patients were followed up for an average of 28.9 ± 15.0 months (95% CI, 25.7–32.1 months). Postoperative complications occurred in 6 patients (7.5%), including 4 cases (5%) of lower limb vein thrombosis, 1 case (1.3%) of surgical site infection, and 1 case (1.3%) of acute periprosthetic joint infection, which were treated with debridement, antibiotics and implant retention (DAIR). No prosthesis loosening was found at the end of the follow-up period. Both the NRS score and KSS improved after the operation (*P* < 0.001) (Table 1). The average degree of varus correction was 4.0 ± 3.6° (95% CI, 3.2–4.7°).

**Table 1:** Preoperative and postoperative demographics of 87 cases of unicompartmental arthroplasty knee (UKA)

	Preoperation	Postoperation	*P* Value
NRS score (median (LQ, UQ))	6.0 (5.0, 7.0)	1.0 (0, 3.0)	< 0.001
KSS (mean ± SD)	113.0 ± 27.2	167.2 ± 23.9	< 0.001
Knee score (median (LQ, UQ))	58.0 (53.0, 70.0)	94.0 (84.0, 99.0)	< 0.001
Functional score (median (LQ, UQ))	50.0 (45.0, 60.0)	80.0 (60.0, 90.0)	< 0.001
FTA (°) (median (LQ, UQ))	181.0 (178.0, 184.0)	176.0 (175.0, 178.0)	< 0.001

*NRS* Numerical Rating Scale, *LQ* lower quartile, *UQ* upper quartile, *KSS* Knee Society Score, *SD* standard deviation, *FTA* femorotibial angle.

### Predictors of KSS improvement

**Fig. 2:**
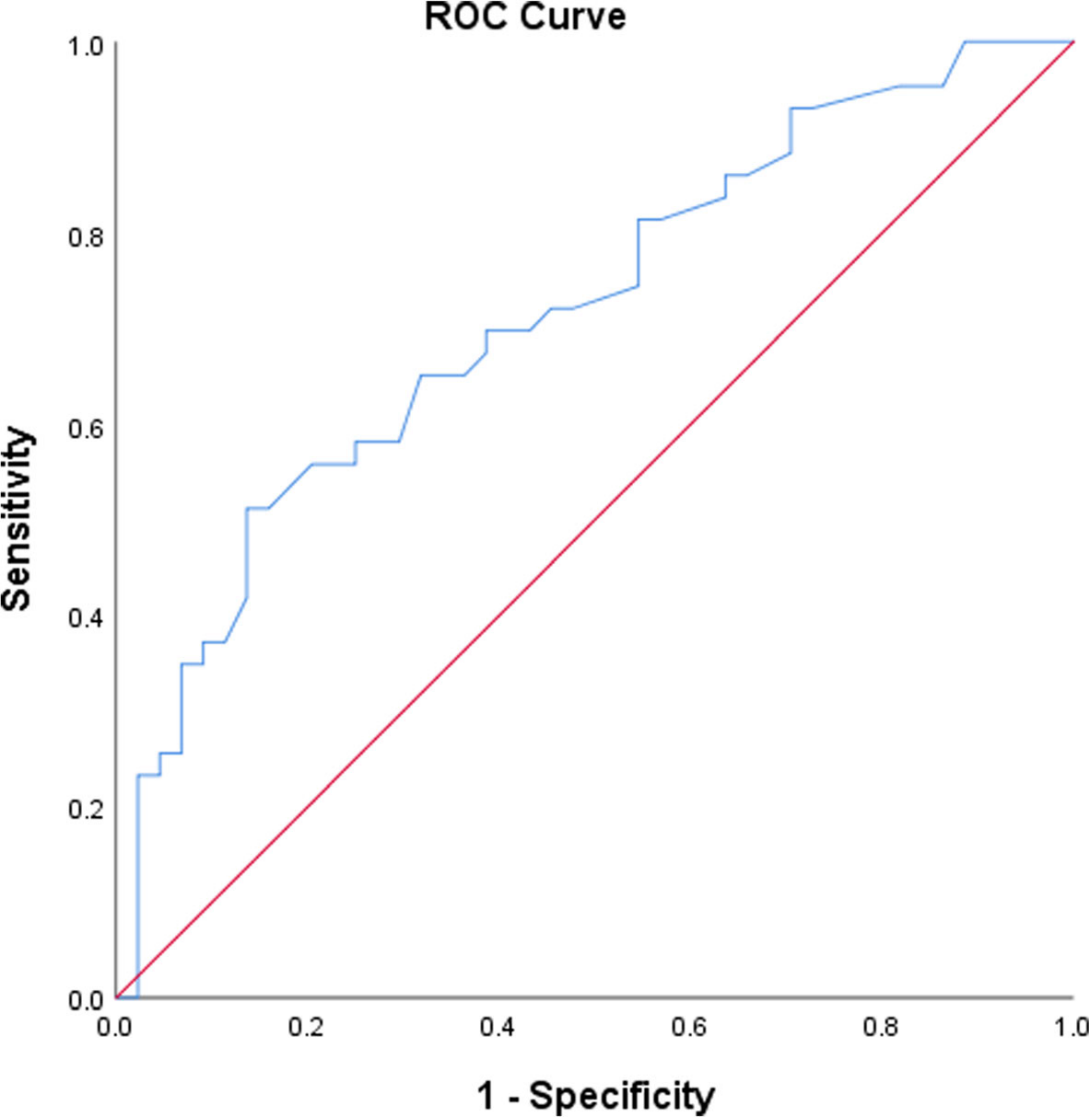
The ROC curve of the combined predictor of KSS improvement. The new combined predictor combined the time since operation (TSO) with the angle E, and the area under the ROC curve was 0.717

The 87 cases treated by UKA were ranked in terms of postoperative improvement in KSS and divided into a less-KSS-improvement group (43 cases) and a more-KSS-improvement group (44 cases). The average KSS improvement was 28.7 ± 20.2 (95% CI, 22.4–34.9) in the less-KSS-improvement group and 79.2 ± 20.1 (95% CI, 73.1–85.3) in the more-KSS-improvement group. There were significant differences in the KSS improvement between the two groups (*P* = 0.000). The two groups were compared in terms of 16 variables, including age, sex, BMI, operated side, SONK, preoperative complications, preoperative NRS, preoperative FTA, operative duration, angle A, angle B, angle E, angle F, prosthesis overhang, prosthesis loosening and TSO. The preoperative NRS, angle E and TSO were found to be significantly different between the two groups (Table 2). Multivariate logistic regression was used to analyze the above 3 potential predictors, and angle E [OR = 3.284 (95% CI, 1.170–9.218, *P* = 0.024)] and TSO [OR = 1.044 (95% CI, 1.011–1.078, *P* = 0.009)] were found to be predictors of KSS improvement (Table 3). The two predictors were combined and included as new predictors in the ROC analysis, and the area under the curve (AUC) was 0.717 (Fig. 2).

**Table 2:** Clinical characteristics of the more-KSS-improvement group and the less-KSS-improvement group

	More-KSS-improvement Group	Less-KSS-improvement Group	*P* Value
Age (year) (mean ± SD)	64.7 ± 7.5	64.3 ± 7.4	0.769
Sex (female:male)	12:32	13:30	0.816
BMI (kg/m^2^) (mean ± SD)	26.4 ± 3.4	25.8 ± 4.4	0.544
Operated side (left:right)	23:21	26:17	0.519
SONK (yes:no)	10:34	5:38	0.256
Preoperative complications (yes:no)	20:24	20:23	1.000
Preoperative NRS score (median (LQ, UQ))	6.0 (6.0, 8.0)	6.0 (5.0, 7.0)	0.020
Preoperative FTA (°) (mean ± SD)	180.2 ± 3.5	181.4 ± 3.6	0.105
Angle A (ED:AD)	0:44	0:43	–
Angle B (ED:AD)	1:43	4:39	0.202
Angle E (ED:AD)	8:36	17:26	0.034
Angle F (ED:AD)	0:44	3:40	0.116
Prosthesis overhang (yes:no)	2:42	3:40	0.676
Prosthesis loosening (yes:no)	0:44	0:43	–
Operative duration (min) (mean ± SD)	105.7 ± 21.7	114.9 ± 28.6	0.097
TSO (month) (mean ± SD)	30.6 ± 13.2	39.2 ± 16.1	0.008

*KSS* Knee Society Score, *SD* standard deviation, *BMI* body mass index, *SONK* spontaneous osteonecrosis of the knee, *NRS* Numerical Rating Scale, *LQ* lower quartile, *UQ* upper quartile, *FTA* femorotibial angle, *ED* excessive deviation, *AD* allowable deviation, *TSO* time since operation.

**Table 3:** Multivariate Logistic regression results of KSS improvement

	OR	95% CI	*P* Value
Angle E	3.284	1.170–9.218	0.024
TSO	1.044	1.011–1.078	0.009

*KSS* Knee Society Score, *OR* odds ratio, *CI* confidence interval, *TSO* time since operation.

### Predictor of patient satisfaction

**Fig. 3:**
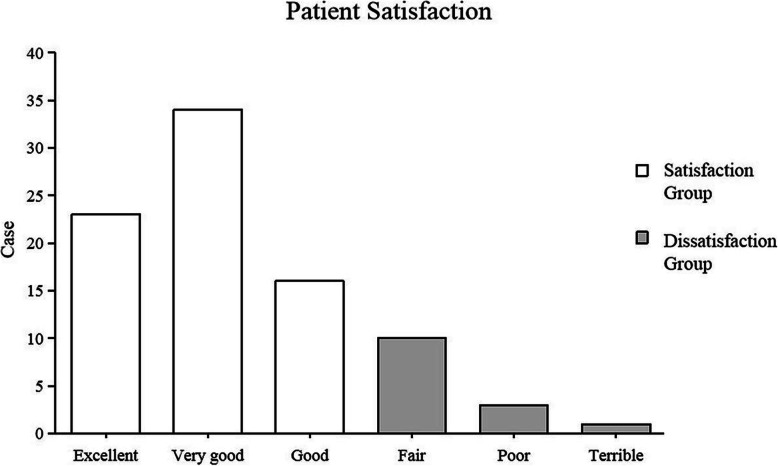
Patient satisfaction at the last follow-up. Of the 87 cases, 23 (26.4%) felt excellent, 34 (39.1%) felt very good, 16 (18.4%) reported good, 10 (11.5%) fair, 3 (3.4%) poor and 1 (1.1%) terrible result

**Fig. 4:**
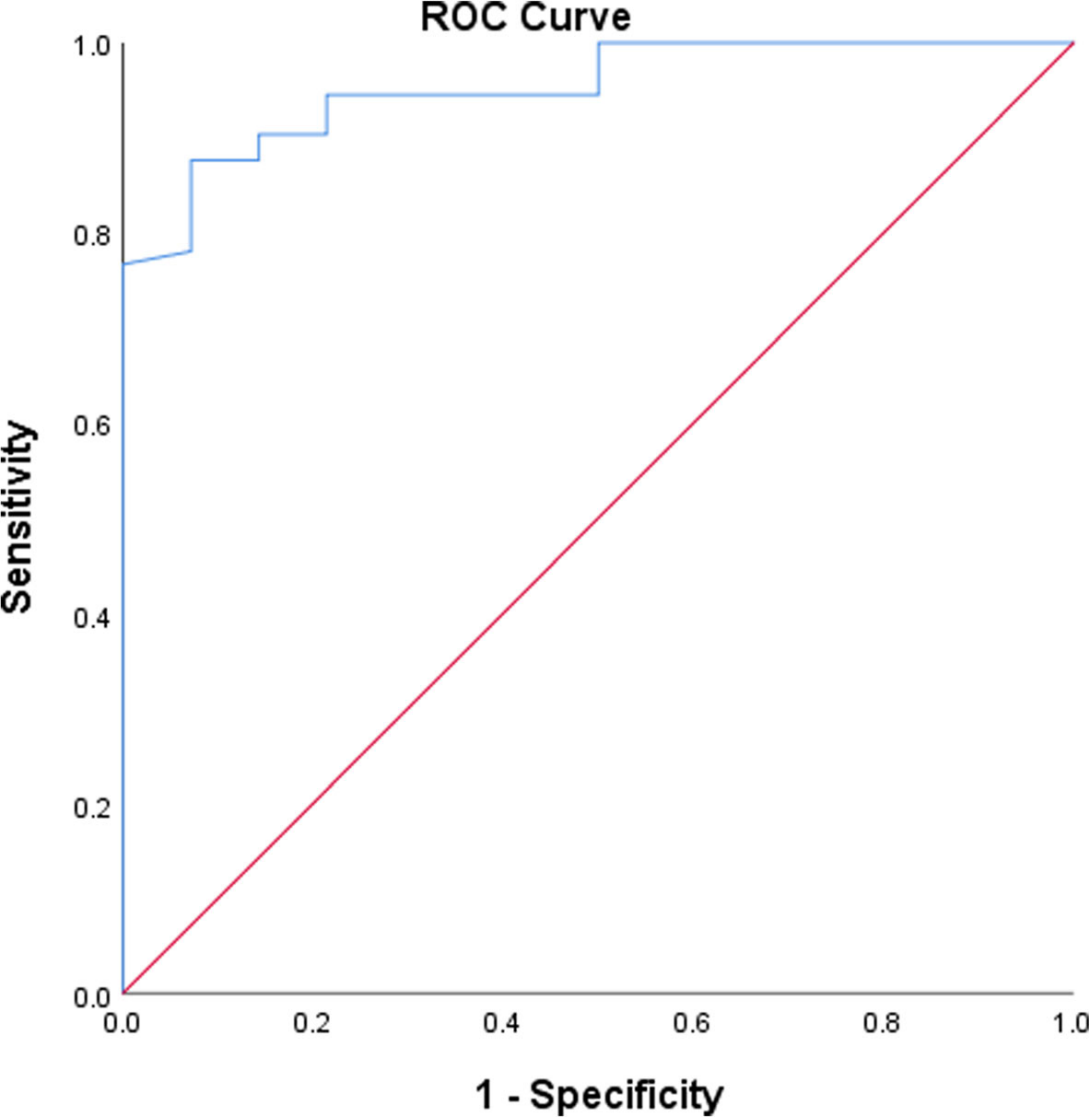
The ROC curve of the combined predictor of patient satisfaction. The new predictor combined the postoperative KSS with the angle E, and the area under the ROC curve was 0.953

At the last follow-up, the level of satisfaction among the 87 cases was as follows: patient satisfaction was excellent in 23 cases (26.4%), very good in 34 (39.1%) cases, good in 16 (18.4%) cases, fair in 10 (11.5%) cases, poor in 3 (3.4%) cases and terrible in 1 (1.1%) case. There were 73 cases (83.9%) in the satisfaction group and 14 cases (16.1%) in the dissatisfaction group (Fig. 3). Comparison was made between the two groups in terms of 28 variables, including age, sex, BMI, operated side, operative duration, SONK, preoperative complications, preoperative NRS, postoperative NRS, NRS score improvement, preoperative knee score, preoperative function score, preoperative KSS, postoperative knee score, postoperative function score, postoperative KSS, KSS improvement, preoperative FTA, postoperative FTA, varus correction, postoperative complications, angle A, angle B, angle E, angle F, prosthesis overhang, prosthesis loosening and TSO. The results showed that a total of 7 variables, including postoperative NRS, NRS score improvement, postoperative knee score, postoperative function score, postoperative KSS, KSS improvement and angle E, differed significantly between the two groups (Table 4). Multivariate Logistic regression was used to analyze the 7 potential predictors selected by univariate analysis, and the result showed that the postoperative KSS [OR = 0.880 (95% CI, 0.817–0.949, *P* = 0.001)] and angle E [OR = 7.723 (95% CI, 1.198–49.764, *P* = 0.032)] were predictors of patient satisfaction (*P* = 0.001; *P* = 0.032) (Table 5). When the two predictors were combined as a new predictor, the AUC of the ROC curve was 0.953 (Fig. 4). In addition, 25 cases (28.7%) had excessive deviation in angle E. Of them, 18 (40.9%) were included in the 44 cases in the early stage of the study, and 7 (16.3%) were included in the 43 cases in the late stage. The cases with excessive deviation in angle E in the early stage were significantly more than those in the late stage (*P* = 0.017) 131313.

**Table 4:** Clinical features of the satisfaction group and the dissatisfaction group

	Satisfaction Group	Dissatisfaction Group	*P* Value
Age (year) (mean ± SD)	64.4 ± 7.5	65.1 ± 7.3	0.753
Sex ratio (female:male)	20:53	5:9	0.532
BMI (kg/m^2^) (mean ± SD)	26.0 ± 3.9	26.6 ± 3.6	0.580
Operated side (left:right)	41:32	8:6	1.000
Operative duration (min) (mean ± SD)	108.5 ± 24.6	119.2 ± 30.0	0.154
SONK (yes:no)	12:61	3:11	0.702
Preoperative complications (yes:no)	33:40	7:7	0.777
TSO (months) (mean ± SD)	34.6 ± 15.6	36.1 ± 14.1	0.746
Preoperative NRS score (median (LQ, UQ))	6.0 (5.0, 7.0)	6.0 (6.0, 7.0)	0.605
Postoperative NRS score (median (LQ, UQ))	1.0 (0, 2.0)	3.0 (2.75, 5.0)	< 0.001
NRS score improvement (median (LQ, UQ))	5.0 (4.0, 6.0)	3.0 (2.0, 4.0)	0.001
Preoperative KSS (mean ± SD)	113.9 ± 28.5	108.3 ± 19.4	0.374
Preoperative knee score (mean ± SD)	58.5 ± 16.0	60.8 ± 10.8	0.608
Preoperative function score (mean ± SD)	55.4 ± 18.5	47.5 ± 10.5	0.126
Postoperative KSS (mean ± SD)	173.7 ± 18.8	133.2 ± 18.4	< 0.001
Postoperative knee score (median (LQ, UQ))	95.0 (85.0, 99.0)	80.0 (62.75, 89.0)	< 0.001
Postoperative function score (median (LQ, UQ))	80.0 (70.0, 90.0)	60.0 (50.0, 60.0)	< 0.001
KSS improvement (mean ± SD)	59.9 ± 30.0	24.9 ± 29.1	< 0.001
Preoperative FTA (°) (mean ± SD)	180.6 ± 3.6	181.6 ± 3.5	0.319
Postoperative FTA (°) (median (LQ, UQ))	176 (175, 178)	177 (176, 178)	0.240
Varus correcting (°) (mean ± SD)	3.9 ± 3.6	4.4 ± 3.6	0.657
Angle A (ED:AD)	0:73	0:14	–
Angle B (ED:AD)	3:70	2:12	0.181
Angle E (ED:AD)	17:56	8:6	0.020
Angle F (ED:AD)	2:71	1:13	0.413
Prosthesis overhang (yes:no)	4:69	1:13	1.000
Prosthesis loosening (yes:no)	0:73	0:14	–
Postoperative complications (yes:no)	5:68	1:13	1.000

*SD* standard deviation, *BMI* body mass index, *SONK* spontaneous osteonecrosis of the knee, *TSO* time since operation, *NRS* Numerical Rating Scale, *LQ* lower quartile, *UQ* upper quartile, *KSS* Knee Society Score, *FTA* femorotibial angle, *ED* excessive deviation, *AD* allowable deviation.

**Table 5:** Multivariate Logistic regression results of patient satisfaction

	OR	95% CI	*P* Value
Postoperative KSS	0.880	0.817–0.949	0.001
Angle E	7.723	1.198–49.764	0.032

*KSS* Knee Society Score, *OR* odds ratio, *CI* confidence interval.

## Discussion

This retrospective study reported the midterm outcomes of Oxford UKAs performed by the same orthopedic surgeon in our medical institution from November 2013 to December 2018. KSS improvement was used as the main objective evaluation index of outcomes, while patient satisfaction was selected as the subjective index, and the predictors of these two indexes were identified.

In our series, both the NRS scores and KSS significantly improved at the last follow-up compared those before the operation. Heyse et al [18] reported 223 patients who underwent UKA and found that the average age was 53.7 years, the average follow-up period was 10 years, the knee score was 94.3 ± 7.8, and the functional score was 94.9 ± 6.8. The knee score was similar to the finding in our study (median score of 94), and the functional score was higher than that in our study (median score of 80). The discrepancy in the functional score might be ascribed to the fact that the patients included in the present study were older (64.5 years vs. 53.7 years) and had performed less intense physical activities. In this study, the NRS score significantly improved after than before the operation. Hoorntje et al [19] found that the NRS scores of outpatients who underwent UKA significantly decreased 3 months after surgery, which was consistent with our results, indicating that UKA can effectively achieve short-term pain relief after surgery.

Due to wear of the articular cartilage of the medial compartment, the medial joint space of the knee becomes narrow, and the FTA increases in patients with medial knee OA. In this study, the preoperative FTA and the postoperative FTA were significantly different (181° vs. 176°, *P* = 0.000), indicating that UKA can correct varus deformities to a certain extent. However, excessive correction of a varus deformity may lead to an excessive load on the lateral compartment. It can also lead to accelerated deterioration of OA in the lateral compartment, which is one of the main reasons for UKA revision [20, 21]. Vasso et al [22] showed that varus angles less than 7° after UKA did not affect the mid- to long-term outcomes of medial UKA, and the results were even better in these patients than in those whose varus deformities were completely corrected. The multivariate analyses in our study also showed that whether the FTA was corrected did not hinder the symptom improvement in our series. Therefore, surgeons performing UKA should focus on how to place the prosthesis in the correct position rather than adjusting the limb alignment.

In our study, the positioning of the implants was evaluated according to the recommendations of the implant manufacturer [17]. Our study showed that excessive deviation in angle E occurred in a large proportion of patients (28.7%), most of whom had excessive varus angles. Most of these patients underwent UKA in the early stage of this study (40.9% vs. 16.3%, *P* = 0.017). Previous studies [23] have pointed out that there is a learning curve in how to perform an Oxford UKA, and UKAs performed at an earlier point in time were associated with a poorer survival rate of the prosthesis, a longer operative duration, more blood loss and a poorer postoperative Hospital for Special Surgery (HSS) knee score. In addition, it has been demonstrated that surgeons’ skill in performing the operation improved only after having performed at least 25 procedures. Therefore, the excessive deviation in angle E, possibly caused by poor positioning of the prosthesis, might be attributed to the inexperience on the part of the surgeon in the early stage. In addition, this study showed that angle E could be used to predict surgical efficacy (KSS improvement) and patient satisfaction, possibly because a deviation in the prosthesis position leads to a change in the alignment and in the load on the limbs, eventually causing pain and other problems [24]. Chatellard et al [25] pointed out that the varus angle of the tibial prosthesis used in UKA should be less than 6° (physiological varus 3° and prosthesis varus 3°) on the coronal plane. Otherwise, the survival rate of the prosthesis will decrease. However, no prosthesis loosening was observed during the follow-up period in this study, which might be related to the relatively shorter follow-up time.

Previous studies reported that the satisfaction rate of UKA ranged from 73 to 96.4% [26–28], and there existed significant differences among these studies. The discrepancies across the studies might result from differences in gender, race, and preoperative mental health of and type of prosthesis used by the patients, and even differences in the methods used for evaluating patient satisfaction [29–31]. The authors of this study attempted to identify the independent predictors of patient satisfaction. However, no demographic characteristics, operative information, or preoperative scores were found to be predictors. Therefore, it was difficult to predict patient satisfaction by any single demographic, surgical or preoperative factor. However, among all the postoperative objective indicators included in this study, both the postoperative KSS and angle E were found to be the predictors of patient satisfaction (*P* = 0.001; *P* = 0.032). OR showed that for every 10-point reduction in the postoperative KSS, the risk of patient dissatisfaction increased by 2.59 times, and for cases with excessive deviation in angle E, the risk of patient dissatisfaction rose by 6.723 times. Therefore, it is reasonable to believe that the correct prosthesis position can improve the postoperative KSS and thus improve patient satisfaction.

This study had some limitations. First, the total number of cases included in this study was small (87 cases), resulting in a small sample size in each group after grouping. Second, all the data in this study were from a single institution, and all UKAs were performed by the same surgeon and his assistants. However, our institution is a regional medical center with a large operation volume, and the results can be, to certain degree, generalized to other population. A multicenter study is warranted to increase the generality of the conclusions. Third, some of the patients in this study were followed up for a short time, which may affect assessment of the long-term efficacy, but all patients completed a follow-up after at least 1 year (12–72 months), and the average follow-up time was 28.9 ± 15.0 months. Finally, all patients in this study were treated with an Oxford unicompartmental knee prosthesis, which has a typical mobile-bearing design. Therefore, the conclusions of this study might not applicable to other types of prostheses. Additional studies are needed to compare the differences between the fixed- and mobile-bearing designs.

## Conclusion

The Oxford UKA can significantly improve both the NRS score and KSS, and it can also correct varus deformities. A shorter TSO and smaller angle E are predictors of greater KSS improvement. In addition, a higher KSS and smaller angle E are predictors of higher patient satisfaction.

## Data Availability

The datasets used and/or analysed during the current study are available from the corresponding author on reasonable request.

## Abbreviations

UKA: Unicompartmental knee arthroplasty
HTO: High tibial osteotomy
TKA: Total knee arthroplasty
OA: Osteoarthritis
SONK: Spontaneous osteonecrosis of the knee
NRS: Numerical Rating Scale
KSS: Knee Society Score
FTA: Femorotibial angle
BMI: Body mass index
TSO: Time since operation
